# FEASIBILITY OF A TELEHEALTH-DELIVERED LIFESTYLE INTERVENTION FOR LATE-MIDLIFE LATINOS LIVING IN RURAL CALIFORNIA

**DOI:** 10.1093/geroni/igz038.511

**Published:** 2019-11-08

**Authors:** Stacey L Schepens Niemiec, Jeanine Blanchard, Cheryl Vigen, Jenny Martínez, Mike Carlson

**Affiliations:** 1 University of Southern California Chan Division of Occupational Science and Occupational Therapy, Los Angeles, California, United States; 2 Jefferson College of Rehabilitation Sciences Department of Occupational Therapy, Philadelphia, Pennsylvania, United States

## Abstract

Late-midlife Latinos (50–64 years old) living in rural regions experience significant health disparities, oftentimes exacerbated by limited access to healthcare services. In a previous pilot study, we observed psychosocial, behavioral, and cardiometabolic health improvements sustained over 12-months in late-midlife Latinos who participated in ¡Vivir Mi Vida! (¡VMV!), a culturally tailored lifestyle intervention led by a Latino community health worker (promotor) and occupational therapist team. For the present study, we assessed the feasibility of telehealth-delivered ¡VMV! modules. Participants (N=10) received an abbreviated three-week version of ¡VMV! consisting of an in-person promotor-led orientation and two one-hour telehealth sessions. Telehealth opinions/experiences were assessed at baseline and follow-up using study-specific questionnaires and by interview. Patient-identified health quality was measured pre-post intervention using the Measure Yourself Medical Outcome Profile (MYMOP2) and a single-item stress index. Participants generally agreed that telehealth session quality was equivalent to in-person sessions, and demonstrated confidence in their ability to communicate freely with the promotor and actively participate in telehealth sessions. We observed significant improvements in nearly all MYMOP2 components and a trend in stress reduction. The intervening promotor reflected that telehealth ¡VMV! extended healthcare to patients impacted by risk factors such as geographic isolation, lack of available services, and hesitancy to access in-person services due to fear of discrimination or deportation. Participants provided highly positive feedback, highlighting the practicality and convenience of the telehealth program. Feasibility of delivering ¡VMV! via telehealth to late-midlife rural-dwelling Latinos and its potential for positive effect was supported.

